# *In vivo* Modeling and Molecular Characterization: A Path Toward Targeted Therapy of Melanoma Brain Metastasis

**DOI:** 10.3389/fonc.2013.00127

**Published:** 2013-05-31

**Authors:** Avital Gaziel-Sovran, Iman Osman, Eva Hernando

**Affiliations:** ^1^Interdisciplinary Melanoma Cooperative Group, NYU Cancer Institute, NYU Langone Medical Center, New York, NY, USA; ^2^Department of Pathology, NYU School of Medicine, New York, NY, USA; ^3^Ronald Perelman Department of Dermatology, NYU School of Medicine, New York, NY, USA

**Keywords:** melanoma brain metastasis, melanoma, brain metastasis, brain tropism, therapy-related, animal models, metastasis

## Abstract

Brain metastasis (B-Met) from melanoma remains mostly incurable and the main cause of death from the disease. Early stage clinical trials and case studies show some promise for targeted therapies in the treatment of melanoma B-Met. However, the progression-free survival for currently available therapies, although significantly improved, is still very short. The development of new potent agents to eradicate melanoma B-Met relies on the elucidation of the molecular mechanisms that allow melanoma cells to reach and colonize the brain. The discovery of such mechanisms depends heavily on pre-clinical models that enable the testing of candidate factors and therapeutic agents *in vivo*. In this review we summarize the effects of available targeted therapies on melanoma B-Met in the clinic. We provide an overview of existing pre-clinical models to study the disease and discuss specific molecules and mechanisms reported to modulate different aspects of melanoma B-Met and finally, by integrating both clinical and basic data, we summarize both opportunities and challenges currently presented to researchers in the field.

## Brain Metastasis from Melanoma – A Clinical Challenge

Brain metastasis (B-Met) occurs in 5–15% of all melanoma patients and is the cause of death in half of metastatic melanoma patients (Johnson and Young, 1996; Sampson et al., 1998; Davies et al., 2011). Disseminated melanoma cells are able to extravasate through the highly restrictive blood brain barrier (BBB) and mostly inhabit the parenchyma, with less frequent leptomeningeal or cerebral spinal fluid (CSF) metastasis.

Currently, treatments for B-Met are determined by their number, anatomic location, surgical risk, systemic disease burden, and leptomeningeal involvement. Patients with a limited number of resectable B-Met may undergo surgical resection or stereotactic radiosurgery. These procedures appear to prolong survival in a subset of patients as reported by retrospective analyses (Lonser et al., 2011; Salvati et al., 2012). Patients with inoperable disease are usually treated with whole-brain radiation therapy (WBRT) or chemotherapy such as temozolomide (Eichler and Loeffler, 2007). Response rates to single-agent chemotherapy are <10%, and treatment simply attempts to slow disease progression (Ewend et al., 2001; Agarwala et al., 2004; Eichler and Loeffler, 2007). It is becoming clearer that the genetic background of a certain patient (i.e., germline mutations) or a tumor should dictate its treatment regimen, and that targeted therapy against these tumor-specific alterations (if available) may be more efficacious. In the case of familial melanoma, germline inactivating mutations in the *CDKN2A/B* locus (mainly p16 and p14) are common (Straume et al., 2002; Gast et al., 2010), leading to aberrant CDK4/cyclin D activity that drives melanoma cell cycle progression. It is plausible that germline mutations contribute to tumor progression by affecting non-melanocytic tissues as well and by that, affecting metastatic potential. For example, certain mutations may affect blood vessels permeability, predisposing patients to increased metastatic spread. The systemic effects of prevalent germline mutations in cancer patients may prove relevant for the development of future tailored personalized medicine. On the other hand, prevalent somatic mutations in melanoma are the subject of intense studies. More than 50% of metastatic melanoma tumors harbor an activating mutation in codon 600 of the *BRAF* gene (V600E or, to a lesser extent, V600K) (Davies et al., 2002). Recently, selective *BRAF* inhibitors such as PLX4032 (vemurafenib) and GSK2118436 (dabrafenib) have shown clinical efficacy in *BRAF* mutant metastatic melanoma patients (Flaherty et al., 2010) with significant tumor regression in approximately 60% of patients (Flaherty et al., 2010). Clinical trials using *BRAF* inhibitors to treat patients with melanoma B-Met were initiated recently with promising results despite the small sample size. A phase I study tested the effects of dabrafenib in 10 patients with untreated and asymptomatic B-Met. Nine of ten patients displayed reductions in size of brain lesions (Falchook et al., 2012). In addition, an ongoing phase II trial is designed to assess the efficacy, pharmacokinetics, safety, and tolerability of dabrafenib administered to a large cohort of subjects with *BRAF V600E/K* mutation-positive B-Met (ClinicalTrials.gov Identifier: NCT01266967). However, resistance to the *BRAF* inhibitor is already evident. In part, this phenomenon is attributed to addiction or functional redundancy within the MAPK pathway, which likely buffers the impact of a single gene/target modification on the malignant process (Johannessen et al., 2010; Nazarian et al., 2010). Moreover, Poulikakos et al. (2011) have identified an additional resistance mechanism in which a splicing variant of mutated *BRAF* that lacks the region encompassing the RAS-binding domain, showed enhanced dimerization in vemurafenib-treated cells.

Another promising, potent agent used lately in late stage melanoma patients is ipilimumab, a monoclonal antibody against the CTLA-4 molecule expressed mainly on regulatory T cells. This antibody blocks CTLA-4 signaling that acts as an immune checkpoint to inhibit T-cell activation [reviewed in (Melero et al., 2007)]. The use of ipilimumab improved overall survival with 10.9% of patients exhibiting complete response, with mostly reversible adverse effects (Hodi et al., 2010) in around 15–20% of patients. Recent reports have suggested that ipilimumab can promote the regression of melanoma B-Met. Case studies reported that ipilimumab significantly benefited individuals with central nervous system (CNS) metastasis (Hodi et al., 2008; Schartz et al., 2010). In a phase II trial of 72 patients with B-Met treated with ipilimumab, 18% of participants that had asymptomatic B-Met and were not treated previously with steroids achieved disease control (partial response or stable disease). The study revealed long-term survival rates comparable to those seen in patients without B-Met, with approximately one-third of patients alive at 12 months. Patients treated with steroids did not show similar responses (Margolin et al., 2012).

The progression-free survival, for both ipilimumab and dabrafenib/vemurafenib-treated patients, although significantly improved, is still very short. Nevertheless, these studies showing unprecedented efficacy against melanoma B-Met exemplify that targeted therapy could be key to the eradication of these highly aggressive metastases.

## Why do Melanomas Metastasize to the Brain?

The concept that metastases arising in different locations in the body carry site-specific characteristics that facilitate tissue colonization is a subject of intense research in various types of cancers. Several studies over the past few years were dedicated to elucidate the molecular and cellular basis of melanoma B-Met, using both experimental and pre-clinical models for this condition.

Interestingly, when melanoma becomes metastatic, it has the highest risk among all tumors for B-Met development with 44–64% of patients (Davies et al., 2011). Moreover, in melanoma patients, a higher proportion of B-Met represent the only site of metastatic disease compared to other solid tumors that frequently metastasize to the brain (Thompson et al., 2004). Strikingly, in a retrospective analysis of more than 2000 melanoma patients our group showed that 36% of melanoma B-Met represent the first and isolated site of metastasis (Ma et al., 2012). Primary tumors of patients from this subgroup displayed distinct clinicopathological features with thinner (mostly stage 1), non-mitotic lesions. Another study by our group of 900 primary melanoma patients showed that location of the primary tumor on the head and neck was an independent predictor of B-Met (Zakrzewski et al., 2011). However, the correlation between anatomical site and B-Met does not hold when analyzing only tumors with B-Met as first isolated site (Ma et al., 2012), suggesting that the predilection to metastasize to the brain is already molecularly “encoded” in some primary melanomas that represent a distinct clinicopathological and possibly molecular entity. It was hypothesized that the high CNS involvement of melanoma may be due to a “homing” effect, since melanocytes and neuronal subpopulations such as glial cells and sensory neurons share a common neural crest progenitor (Herlyn et al., 2000). However, this hypothesis has not yet been yet thoroughly investigated experimentally.

From a molecular point of view it is imperative to ask whether a specific set of conditions need to occur in order for melanoma cells to seed and proliferate in a certain tissue. Multiple studies, mainly in the context of breast cancer, demonstrated how metastasis to different sites involves unique programs that facilitate tumor cell seeding and proliferation within the myriad of specialized cell types and extracellular matrices of the foreign tissue (Padua et al., 2008; Bos et al., 2009; Zhang et al., 2009a). Organ specificity can also be achieved by differential expression of molecules on resident cells of the invaded tissue. For example, the adhesion molecule Lu-ECAM-1 was reported to be specifically expressed on distinct branches of lung blood vessels, facilitating the arrest and binding of melanoma cells with higher affinity to it (Zhu et al., 1991). As for tropism of cancer cells to the brain, a study by Weiss (1992) estimated that the arrival of 66% of hematogenous B-Met may be explained by blood circulation while the remaining metastases may reflect site specificity.

## *In vivo* Models of Melanoma Brain Metastasis

Several groups have reported the development and use of *in vivo* models of melanoma B-Met (Fujimaki et al., 1996; Yano et al., 2000; Küsters et al., 2003; Xie et al., 2006; Huang et al., 2008; Zhang et al., 2009b) (summarized in Table 1). Nonetheless, there are considerable shortcomings in most of them. The ‘spontaneous’ B-Met model induced through subcutaneous transplantation of tumor cells in the flank allows sufficient time for primary tumor cells to disseminate and establish distant metastases (Cruz-Munoz et al., 2008). In such model, a melanoma cell line was used to generate a systemic metastatic disease in NOD/SCID mice. Mice were then subjected to a metronomic chemotherapy and surviving mice developed spontaneous B-Met. Cell lines established from B-Met were then proven to metastasize to the brain parenchyma efficiently and with shorter latency. This model recreates the multiple sequential steps that are associated with the metastatic cascade, making it closely resembled to the clinical disease. However, the long latency period needed for metastatic disease in the brain to become evident, the relatively low incidence, and the limited number of syngeneic and xenograft spontaneous B-Met models available makes this approach less appealing when compared to other models.

**Table 1:** ***In vivo*. models of melanoma brain metastasis**.

Model	Technique	Advantages	Limitations
Spontaneous brain metastasis (Cruz-Munoz et al., 2008; Cruz-Muñoz et al., 2012)	Subcutaneous implementation of pre-selected clones followed by tumor resection. Metastatic disease in the brain is allowed to occur spontaneously from metastasizing cells leaving the subcutaneous implementation site	Recreates the multiple sequential steps that are associated with the metastatic cascade, making it closely resembled to the clinical disease Suitable for pre-clinical testing of adjuvant therapies	Relatively low throughput Very long latency period needed for metastatic disease in the brain to become evident Relatively low incidence Limited number of available pre-selected cell lines to be used
Intra-carotid injection (Fujimaki et al., 1996; Yano et al., 2000; Xie et al., 2006; Huang et al., 2008; Zhang et al., 2009b)	Cells are injected into the internal carotid artery	Allows for controlled delivery of cancer cells Offers a short time for metastatic disease to manifest Availability of many well-characterized cell lines	Technically challenging Does not reflect the complete series of events involved in the metastatic process Extremely short latency between tumor induction and mortality Mostly leptomeningeal metastases are formed
Intra-cardiac injection (Izraely et al., 2012; Tekle et al., 2012; Morsi et al., 2013; Sundstrøm et al., 2013)	Cells are injected into the left ventricle of the heart	Relatively high-throughput Recapitulates most relevant stages of the metastatic spread to the brain Technically feasible May produce parenchymal lesions Reasonable latency between inoculation to appearance of brain metastasis – may be used for pre-clinical testing of adjuvant therapies	Does not reflect the complete series of events involved in the metastatic process Limited number of available pre-selected cell lines to be used
Injection into chick embryo (Busch et al., 2012)	Cells are injected into the rhombencephalic brain vesicle of a 2-day-chick embryo. Two to three days post-injection tumor formation is studied	Fast Controlled delivery of cells May be used with multiple cell lines	Physiological relevance is not yet established Limited to study certain processes such as extravasation and local invasion

Mouse models in which melanoma B-Met is induced through direct injection of cancer cells into the circulation, known as ‘experimental’ models, do not reflect the complete series of events involved in the metastatic process. Nevertheless, they allow for both controlled delivery of cancer cells and a short time for metastatic disease to manifest. These models are particularly suitable to study later stages of B-Met such as seeding and tissue colonization. These characteristics, along with the availability of many well-characterized cell lines, make these models attractive to study B-Met in pre-clinical settings. Notably, nearly all of the experimental melanoma B-Met studies use internal carotid artery injections (Fujimaki et al., 1996; Yano et al., 2000; Küsters et al., 2003; Xie et al., 2006; Huang et al., 2008; Zhang et al., 2009b). This method of tumor induction, although still a commonly used methodology, is time consuming and requires certain level of surgical expertise. In addition, this route of injection proved to be “artificially” invasive, with extremely short latency between tumor induction and mortality, putting its physiological relevance in question. Moreover, the B16 syngeneic cell line used vastly in this model develops exclusively leptomeningeal metastasis, as opposed to the more prevalent parenchymal dissemination. This considerable shortcoming renders the B16 a less clinically relevant model with low translational potential.

Recently, intra-cardiac injection has been established as a less invasive and less technically demanding route of B-Met induction. In these studies, human cells are injected directly into the left ventricle of the heart of immuno-deficient mice to develop a more clinically relevant *in vivo* model. Following this methodology, human melanoma cell lines directly injected in athymic nude (Izraely et al., 2012) or Balb/c mice (Tekle et al., 2012) successfully developed parenchymal lesions. A new model developed recently by Sundstrøm et al. (2013) utilized intra-cardiac injection of melanoma cells labeled with superparamagnetic iron oxide nanoparticles (SPIONs). These cells were effectively visualized by magnetic resonance imaging (MRI) followed by automated analysis. Our group combined ultrasound-guided intra-cardiac injection of melanoma cells as a minimally invasive, high-throughput method of induction, with MRI-assisted tumor segmentation, 3D reconstruction and quantitative volumetric analysis, to precisely map and measure parenchymal B-Mets (Morsi et al., 2013). This approach takes advantage of the paramagnetic nature of melanin, which renders a signal brightening endogenous effect in tracer-free T1-weighted MRI (Isiklar et al., 1995). Importantly, the metastatic pattern observed in both studies resembled the one seen in patients and was highly reproducible. This type of in-depth characterization of the growth pattern of B-Met lesions developing within *in vivo* models, using various imaging techniques, will allow to faithfully assess melanoma brain tropism, seeding and adaptation, study the molecular mechanisms that control these processes, and may be used to test potential therapeutic agents.

Lately, a study by Busch et al., made use of the chick embryo model to study melanoma B-Met. Melanoma cell lines were injected into the rhombencephalic brain vesicle of the 2-day-chick embryo. Two to three days post-injection, tumor formation was studied in serial paraffin sections (Busch et al., 2012). The chick embryo model is inherently limiting in studying crucial stages of melanoma dissemination to the brain but can be exploited to study early phases such as extravasation and local invasion in the brain.

## How do Melanomas Reach and Adapt to the Brain Microenvironment?

The exact sequence of events required by tumor cells for successful colonization of the brain remains obscure. Kienast and colleagues used multiphoton laser scanning real-time microscopy to follow single steps of B-Met formation. Their innovative experimental system enabled them to follow melanoma cells injected into the internal carotid artery arrest at vascular branch points, extravasate early, remain in close and persistent contacts to microvessels, and co-opt the vessel for nutrients. This final step was unique for melanomas that, as opposed to lung cancer-derived cells, did not induce early angiogenesis (Kienast et al., 2010). This particular finding is intriguing and suggests that B-Met originating from different tumor types possess distinct molecular properties and may respond differently to certain therapies and thus, should not necessarily be treated uniformly as one entity.

**Figure 1:**
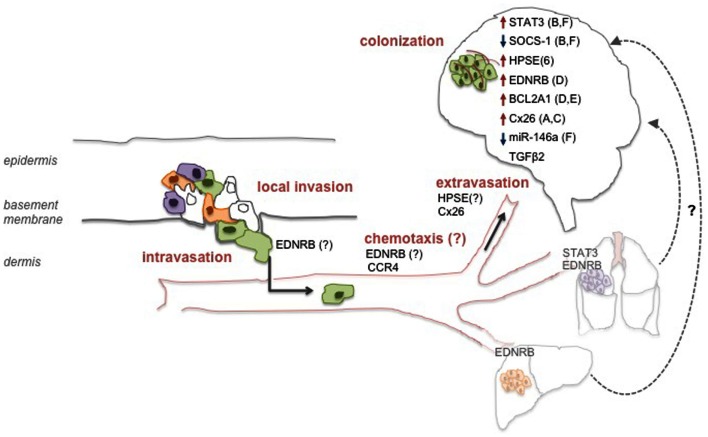
**Molecular determinants of melanoma brain metastasis**. Molecular alterations may occur on all consecutive steps that support the metastatic process of melanoma cells to the brain. Alterations in specific factors may support processes at the primary tumor site and endow a subset of cells with the ability to reach the brain (represented by green cells). Other factors may direct migration to the brain via chemotaxis, or promote adhesion and extravasation through the BBB. Lastly, inside the brain, other factors facilitate several processes that allow the successful colonization of the tissue such as vessel co-option (A), angiogenesis (B), seeding (C), growth (D), survival (E), or invasiveness (F). Although not yet demonstrated for melanoma, it is possible that metastatic spread to the brain may also originate from other visceral metastatic sites such as the lung or the liver (“metastasis of metastasis”).

The multistage process of metastatic spread to the brain requires the involvement and integration of multiple biological events. *In vitro* and *in vivo* models studying the nature of the alterations required for melanoma cells’ tropism to the brain revealed a number of effectors to mediate different aspects of that process. Interestingly, most reports do not claim the alterations found to be exclusive of B-Met. While those are relevant for the elucidation of the mechanisms that govern melanoma B-Met, the discovery of site-specific molecular alterations may be key for the development of potent, site-specific therapy. This approach may be highly beneficial for patients, especially since current data clearly point to a model in which melanoma B-Met are not always a late stage metastatic disease but may also be a unique entity with possibly distinct molecular profile. Below we summarize some of the molecular factors implicated so far in melanoma B-Met models (depicted in Figure 1).

## JAK-STAT

The JAK-STAT pathway, that promotes survival, growth, and angiogenesis, was reported to increase melanoma B-Met mainly via STAT3 activation by phosphorylation, or downregulation of its inhibitor SOCS-1 (Xie et al., 2006; Huang et al., 2008). The main effects observed in both studies were increased expression of MMP-2, bFGF, and VEGF, possibly supporting melanoma cell invasion and angiogenesis. Importantly, STAT3 activation and consequential effects were a more general pro-metastatic phenomenon, not restricted to B-Met. In fact, a recent study showed that melanoma lung metastases exhibited the highest level of p-STAT3 expression and that p-STAT3 expression was not associated with an increased risk of developing B-Met or time to B-Met (Lee et al., 2012). It still remains to be determined whether the effects of SOCS-1 are more specific to B-Met, but since its main downstream target reported in the study was STAT3, a brain metastatic-specific mechanism seems unlikely.

## Heparanase

The enzyme Heparanase (HPSE) degrades heparan sulfate chains of proteoglycans that are known to have multiple functions including maintaining capillaries support or retaining soluble factors (e.g., chemokines). Using a brain slice model it was shown that higher HPSE levels lead to increased invasion of the brain, that was repressed when specific HPSE inhibitors were used (Murry et al., 2006). In support of the role of HPSE in promoting B-Met is a recent study that reported miR-1258 to be a suppressor of breast cancer B-Met through the direct targeting of HPSE (Zhang et al., 2011). Since HPSE is a potent pro-tumorigenic and pro-metastatic agent, its effects might not be confined to brain-specific processes. Still, HPSE role could be more evident in B-Met models and patients’ samples since its activity is essential for the successful extravasation of the blood-borne melanoma metastasis through the heparan sulfate-rich endothelial cell layer. Furthermore, co-incubation of astrocytes with melanoma brain metastatic cells resulted in elevated HPSE activity and markedly increased invasive capacity *in vitro* (Marchetti et al., 2000). This further supports brain-specific activity for this enzyme.

## Endothelin Receptor B

A recent study has implicated Endothelin Receptor B (EDNRB) as a factor that potentially influences brain metastatic potential. Using a pre-clinical model of melanoma B-Met developed by the same group (Cruz-Munoz et al., 2008), the authors showed that EDNRB overexpression enhanced overall metastatic disease, and increased the incidence of spontaneous B-Met. The study showed that the interaction of EDNRB with its ligands caused increased intracranial melanoma growth. Therapeutic treatment by an EDNRB-specific inhibitor translated into improved outcome in mice. This study implicates a protein critical for melanocyte biology in promoting melanoma metastatic potential in general and B-Met in particular (Cruz-Muñoz et al., 2012). Although the pro-metastatic effects were not exclusive to the brain, the authors postulate that the high levels of EDNRB ligands in the brain relative to other organs may explain the overall increased growth within the brain and the increased frequency of B-Met in this study. Importantly, endothelin 3 levels are also high in lung tissue and may be responsible for the increased lung metastasis frequency when EDNRB was ectopically expressed (Fagan et al., 2001). These results are exciting since they exemplify how melanoma metastatic cells are affected by surrounding specific microenvironmental ligands and utilize them for their growth. The successful therapeutic aspect of this study highlights EDNRB as a potential druggable target. Interestingly, EDNRB overexpression was ectopically induced within the implanted tumor in the flank where endothelins are not abundant. Still, EDNRB overexpressing cells metastasized more frequently to the brain. This points to a model in which EDNRB generally facilitates metastatic spread, but its effects are exacerbated in the brain, where its ligands are abundant.

## BCL2A1

A second factor implicated in the same study is the anti-apoptotic protein BCL2A1, which did not affect the incidence of B-Met but facilitated intracranial tumor growth, possibly by enhancing cell survival. Since cells were injected intra-cranially in those experiments, this finding needs to be further investigated for its physiological relevance (Cruz-Muñoz et al., 2012).

## Connexin 26

Connexins have been lately shown to mediate early events in brain colonization using transparent zebrafish and chicken embryo models of B-Met. One study showed that melanoma cells utilize the gap junction protein Connexin 26 (Cx26) to initiate B-Met formation in association with the vasculature. Cx26 silencing or pharmacological inhibition of connexins blocked cell extravasation and blood vessel co-option (Stoletov et al., 2013). The idea that specific connexins mediate cancer metastasis to the brain by increasing gap junction communication with the BBB is intriguing, particularly in the context of previous observations highlighting vessel co-option among the initial steps of brain colonization unique to melanoma (Kienast et al., 2010). Interestingly, a study by Lin et al., reported that activated astrocytes surrounding melanoma B-Met protect them from chemotherapeutic drugs. This chemo-protection was dependent on physical contact and gap junctional communication between astrocytes and tumor cells (Lin et al., 2010). It will be interesting to examine whether the specific silencing of Cx26 will be sufficient to eliminate these chemoprotective effects.

## CCR4

The expression of the chemokine receptor CCR4 was found significantly higher in one melanoma brain metastatic variant compared to the corresponding tumor implanted in the flank (Izraely et al., 2010). The same group has reported that brain-derived soluble factors upregulate the expression of CCR4 in both cutaneous and brain-metastasizing melanoma cells and enhance the migration of the latter, but not that of the cutaneous variants (Klein et al., 2012). These findings support the hypothesis that some alterations may occur early at the primary tumor site where certain clones express molecules that promote spread of melanoma cells to the brain. One can postulate that CCR4 ligands secreted from the brain interact with the CCR4-positive melanoma cells and attract them to the brain. This kind of directed migration was reported previously for breast cancer cells overexpressing CXCR4 that facilitated their transmigration through the brain endothelial cells (Lee et al., 2004).

## TGFβ2

Overexpression of TGFβ2 in mouse melanoma cells increased their ability to seed in the brain parenchyma, suggesting a role for this pathway in determining site specificity in the brain microenvironment (Zhang et al., 2009b). This study illustrates how specific factors may be crucial for B-Met growth and potentially be exploited therapeutically to diminish successful seeding.

## miR-146a

MicroRNAs (miRs) have demonstrated to play critical roles in cancer metastasis including melanoma [reviewed in (Segura et al., 2012)]. miRs emerge as optimal candidates to regulate such a complex and multi-layered process as the metastatic dissemination within the brain due to their ability to concomitantly control multiple targets and thus impact various molecular processes. A recent study found miR-146a to be nearly undetectable in melanoma cells selected to metastasize to brain relative to their parental counterparts. Overexpression of miR-146a suppressed the migratory and invasive capacity of those cells possibly by targeting hnRNPC and increasing β-catenin (Hwang et al., 2012). While the clinical relevance of this finding needs to be further elucidated, ongoing studies focusing on the potential roles of miRNAs in the modulation of melanoma B-Met might provide a deeper understanding of the critical pathways that drive or support this condition.

## BBB Disruptors

Since melanoma B-Mets are blood-borne, cells must extravasate through the highly restrictive BBB. Thus, the integrity of the BBB is essential for the prevention of metastatic infiltration. An *in vitro* model of the BBB demonstrated how melanoma cells are able to penetrate the BBB disrupting major tight junctions molecules such as ZO-1 and Claudin-5, and reducing transendothelial electrical resistance (TEER), an indicator of junctional integrity (Fazakas et al., 2011; reviewed in Wilhelm et al., 2011). The mechanism by which melanoma cells induce endothelial cells junctional degradation is still unclear but the ability of supernatants of melanoma cells to generate similar effects points to the involvement of secreted soluble factors such as proteolytic enzymes mentioned above.

The development and use of models to study melanoma B-Met is yielding potential candidates as regulators of B-Met. However, the physiological relevance of those factors to human disease should be further confirmed to conclusively establish their clinical impact.

## Challenges and Opportunities

In recent years conventional therapeutic regimens are clearing the way for tailored, patient-specific therapy. This approach is aimed to maximize responsiveness to treatment based on the tumor’s genetics while indirectly reducing side effects caused by the administration of ineffective treatments, and sparing the normal cells of the body that do not harbor the same genetic alteration. Some case reports and early phase clinical trials show promise for targeted therapies in the treatment of melanoma patients with B-Met. This is encouraging, especially since those patients have been, thus far, systematically excluded from most clinical trials. Still, current therapies improve overall survival only marginally and there is a pressing need for B-Met-specific treatments. The notion that the predilection to metastasize to the brain is present in melanoma cells possibly already at the time of primary diagnosis provides a unique opportunity to use specific adjuvant therapy to prevent or reduce metastatic dissemination in patients at higher risk of developing B-Met. The characterization of mechanisms that endow cells with brain-specific tropism and colonization is incipient and ought to be thoroughly investigated. This might add another layer of specificity to the treatment regimens patients are offered based on their site of metastatic dissemination.

The development of *in vitro* and *in vivo* models of melanoma B-Met to discover the molecular mechanisms underlying melanoma B-Met has progressed significantly. Molecular alterations most often seen in melanoma B-Met are typically those resulting in: (i) increased BBB permeability (via junctional, adhesion, and proteolytic factors), (ii) increased tropism to brain microenvironment (via chemokine and cytokines signaling), (iii) enhanced survival in the brain (through modulation of pro-proliferative and anti-apoptotic factors). Nevertheless, novel imaging techniques such as multiphoton microscopy may provide better resolution, real-time assessment of the metastatic process in the brain and its modulation by certain molecules or therapies. Data accumulated from current and future experimental and pre-clinical models of melanoma B-Met should be used to develop new site-specific therapies to efficiently target melanoma B-Met. One can envision therapies focusing on preventing the arrival and seeding of melanoma cells to the brain by blocking certain cell surface receptors or secretion of specific proteolytic enzymes. Targeting the specific interactions of melanoma B-Met with resident cells in the brain parenchyma is another good example of future site-specific therapy that may be developed relying on data arising from pre-clinical models. The outstanding question of whether melanoma B-Met is indeed a separate molecular entity remains mostly unanswered. In that regard, generalized use of next-generation deep sequencing of clinical specimens should provide new insights and might alter dramatically our perception of this disease.

## Conflict of Interest Statement

The authors declare that the research was conducted in the absence of any commercial or financial relationships that could be construed as a potential conflict of interest.
